# Risk factors for recurrence after surgical repair of coarctation of the aorta in children: a single-center experience based on 51 children

**DOI:** 10.3389/fcvm.2023.1144755

**Published:** 2023-05-30

**Authors:** Zhenjiang Zhao, Zhengxia Pan, Chun Wu, Jie Tian, Jinjie Qin, Yulin Zhang, Xin Jin

**Affiliations:** ^1^ Department of Cardiothoracic Surgery, Children’s Hospital of Chongqing Medical University, Chongqing, China; ^2^ Ministry of Education Key Laboratory of Child Development and Disorders, Chongqing, China; ^3^ National Clinical Research Center for Child Health and Disorders, Chongqing, China; ^4^ China International Science and Technology Cooperation Base of Child Development and Critical Disorders, Chongqing, China; ^5^ Chongqing Key Laboratory of Pediatrics, Chongqing, China; ^6^ Department of Cardiology, Children’s Hospital of Chongqing Medical University, Chongqing, China; ^7^ Department of Radiology, Children’s Hospital of Chongqing Medical University, Chongqing, China; ^8^ Intelligence Medical of Science and Technology Commission of Chongqing, Chongqing, China

**Keywords:** coarctation of the aorta (COA), congenital heart disease - cardiac, pediatrics - children, risk factor, surgical treatment

## Abstract

**Background:**

Coarctation of the aorta (CoA), is a congenital malformation, often combined with several cardiac abnormalities. At present, the operation effect is satisfactory, but postoperative restenosis is still a matter. Identification of risk factors for restenosis and prompt therapy adjustments may improve patient outcomes.

**Materials and methods:**

A retrospective clinical study of patients under 12 who had CoA repair in 2012–2021, with a randomized cohort population of 475 patients.

**Results:**

A total of 51 patients (M/F: 30/21) with a mean age of 5.33 (2.00–15.00) months and a median weight of 5.60 (4.20–10.00) kg. The mean follow-up was 8.93 (3.77–19.37) months. Patients were divided into 2 groups: no-restenosis (n-reCoA) (G1, 38 patients) and restenosis (reCoA) (G2, 13 patients). ReCoA was defined as a restenosis requiring interventional or surgery or a pressure gradient >20 mmHg at the repair site as reported by B-ultrasound with the presence of an upper and lower limb blood pressure gradient or growing dysplasia. The overall reCoA incidence was 25% (13/51). In multivariate COX regression, smaller preoperative z-score of the ascending aorta (*P* = 0.009, HR = 0.68) and transverse aortic arch (*P* = 0.015, HR = 0.66), arm-leg systolic pressure gradient ≥12.5 mmHg at discharge (*P* = 0.003, HR = 1.09) were independent risk factors for reCoA.

**Conclusion:**

The overall outcome of CoA surgery is successful. Smaller preoperative z-score of the ascending aorta and transverse aortic arch, and an arm-leg systolic pressure gradient ≥12.5 mmHg at discharge increase reCoA risk, and closer follow-up for such patients are required especially within 1 postoperative year.

## Introduction

Coarctation of the aorta (CoA), a congenital abnormality, often occurs in the arterial catheter or arterial ligament region, which has an incidence rate of about 0.287‰ and accounts for 3.57% of all congenital cardiovascular malformations (1). It can be isolated or with additional cardiac abnormalities such as ventricular septal defect and patent ductus arteriosus (2). End-to-end anastomosis (EEA) was first proposed by Crafoord and Nylin in 1945 (3), but with a significant rate of restenosis (reCoA) (4–6). People have used a variety of methods to reduce postoperative mortality and restenosis rate to improve patient survival time and quality, including patch aortoplasty (PAP), subclavian artery valvuloplasty, expanded end-to-end anastomosis (EEEA), end-to-side anastomosis (ESA), and balloon angioplasty. Advancements in science and technology have dramatically reduced mortality (7), therefore reCoA has attracted more focus. Several studies have shown contradictory outcomes on age and weight at the surgical procedure and other variables (8–12). This research aimed to collect data and examine risk factors of reCoA to guide clinical practice.

## Materials and methods

### Study population

This is a retrospective study of patients who underwent CoA surgical procedures at the Children's Hospital of Chongqing Medical University between 2012 and 2021. Patients were eligible if they met the following criteria: (1) age ≤12 years old, (2) had preoperative echocardiography and cardiac CT scan and three-dimensional reconstruction, (3) CoA is the main diagnosis, and (4) successful follow-up after discharge. Evaluate available data (all obtained by gathering clinical records, surgery reports, and discharge records) to detect reCoA risk factors. A total cohort population of 51 people was enrolled randomly from 475 patients. Demographic data (such as gender, age, and weight at the time of surgical procedure), perioperative data [such as whether cardiotonic agents were used before surgery, operation procedures, time of cardiopulmonary bypass (CPB), and so on], and follow-up data were gathered. The surgical treatment was considered a success if patients did not die after the operation, the Doppler pressure gradient across the repair site <20 mmHg during the follow-up, and the blood pressure of the upper limb was lower than that of the lower limb, and no evidence of hypertension. The hypertension diagnostic criteria were based on the China Guidelines for Prevention and Treatment of Hypertension, the systolic blood pressure (mmHg) for boys over 100 + 2× age (years), and girls over 100 + 1.5× age (years), or taking antihypertensive agents. When the z-score of the transverse arch < −2, hypoplastic aortic arch (HAA) can be diagnosed. The pressure gradient obtained by echocardiography varies depending on the location of the constriction. Use echocardiography to determine the flow rate at the constriction, and the pressure was estimated using the simplified Bernoulli equation *P* = 4V^2^. The Children's Hospital of Chongqing Medical University's Ethics Committee authorized the study, and all patients signed the informed consent form.

### Surgical technique

The suitable surgical technique was chosen based on the patient's clinical state, including combined intracardiac malformations, preoperative cuff blood pressure, medical imaging data such as ultrasonic cardiogram and CT scan, and requests of their parents. A median sternotomy method is chosen for patients with intracardiac abnormalities that require simultaneous repair and CPB. A lateral thoracotomy method is chosen for patients with intracardiac abnormalities that can be treated minimally invasively without CPB. EEEA, ESA, and PAP are the three main categories of surgical techniques. The anastomotic strain encountered throughout the procedure determines the use of certain surgical techniques. In general, patients with simple isthmus aortic coarctation are treated with EEEA, whereas those with hypoplastic aortic arch may be treated with ESA and PAP. It should be highlighted that SAR was chosen by two patients. After performing SAR on the two patients during the operation, the surgeon noticed that the invasive systolic blood pressure difference between the brachial artery and the femoral artery had returned to normal (upper limb < lower limb). The surgeon then immediately informed the patients' families of this finding and they decided to change the preoperatively decided surgical plan and adopt SAR.

### Statistical methods

Kolmogorov-Smirnov test examines the normality of continuous variables. Normal distribution variables were shown as mean ± standard deviation (SD), compared by Student's t-test or ANOVA. The skewed distribution variables were shown as medians (P25, P75), compared by the Wilcoxon signed-rank test. Chi-square test, corrected Chi-square test, or Fisher exact test compared rates or component ratios. Echocardiographic data and demographics were potential predictors. Only variables (*P* < 0.2) with univariate COX regression were included in multivariable COX regression operated by stepwise regression. ROC curve was used to determine the best cutoff (Jordan index-based) with the maximum sensitivity and specificity for a critical continuous risk factor. AUC measured accuracy. ReCoA-free survival rate was conducted by Kaplan–Meier curve. The log-rank test compared patient subgroups' differences in freedom from event (reCoA vs. n-reCoA) rates. The statistically significant difference was defined as *P* < 0.05, and all statistical analyses were performed using SPSS26.0 software.

## Results

The study cohort included 51 patients (30 males, 21 females) with a 25% (13/51) incidence of reCoA. The median weight was 5.60 kg (interquartile range, 4.20–10.00 kg) and the mean age was 5.33 months (interquartile range, 2.00–15.00 months). The mean follow-up was 8.93 months (interquartile range, 3.77–19.37 months). Patients were divided into two groups: n-reCoA (G1, 38 patients) and reCoA (G2, 13 patients). Table 1 compares demographics and surgical variables. Concomitant hypoplastic aortic arch appeared to be a reCoA risk factor with 10 patients (26%) in G1 and 8 patients (62%) in G2, respectively (*P* = 0.050). As for gender, 10 males and 3 females had reCoA (*P* = 0.125). And 6 of 26 VSD patients (23%) and 7 of 25 non-VSD patients (28%) had ReCoA (*P* = 0.687). Surgical age shows no significant difference in <1 month compared with the other two groups (*P* = 0.751). Variables including surgical weight and CPB time did not statistically differ (*P* > 0.05).

**Table 1:** Demographic and perioperative variables.

Variable	G1 no-reCoA (*n* = 38)	G2 reCoA (*n* = 13)	*P* value
Age at operation			
<1 mo	6	2	0.751
1–12 mo	22	6	
1–12 yrs	10	5	
Weight at operation (kg)	5.05 (4.07–8.75)	9.00 (4.70–10.00)	0.173
Incision (median sternotomy/lateral chest)	19/19	7/6	0.811
Crossclamp time (min)	43.31 (24.79–83.00)	54.93 ± 7.23	0.681
CPB time (min)	108.49 ± 7.59	117.42 ± 10.28	0.536
Operation time (min)	212.50 (113.75–250.75)	193.62 ± 17.78	0.991
Extubation time (d)	5.50 (4.00–8.50)	6.08 ± 0.65	0.896
ICU time (d)	9.00 (4.00–13.00)	8.04 ± 1.17	0.690
Hospitalization time (d)	25.46 ± 1.42	20.69 ± 1.92	0.079
Congenital heart malformation, *n* (%)			
VSD	20 (53)	6 (46)	0.687
ASD	23 (61)	6 (46)	0.366
PDA	29 (76)	8 (62)	0.502
BAV	3 (8)	2 (15)	0.808
HAA	10 (26)	8 (62)	0.050
Mitral stenosis	3 (8)	0	0.561
PLSVC	3 (8)	2 (15)	0.808
Congenital extracardiac malformation	7 (18)	1 (8)	0.634

Comparison of demographic and surgical variables. Values are shown as mean ± standard deviation (SD), median (P25-P75), or number (percent %). CPB, cardiopulmonary bypass; ICU, intensive care unit; VSD, ventricular septal defect; ASD, atrial septal defect; PDA, patent ductus arteriosus; BAV, bicuspid aortic valve; HAA, hypoplastic aortic arch; PLSVC, persistent left superior vena cava.

**Figure 1:**
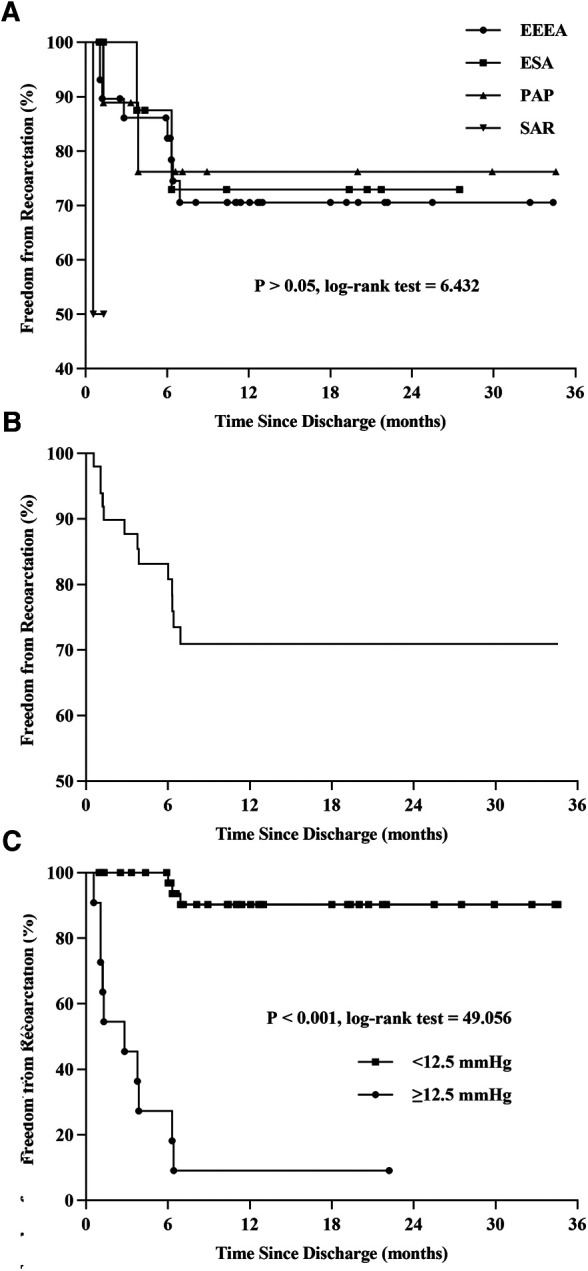
Kaplan-Meier curve. Compare the differences of reCoA among the six surgical options **(A)**. Freedom from reCoA after discharge **(B)**. Patients with a blood pressure gradient ≥12.5 mmHg at discharge have a significantly higher rate of reCoA **(C)**. EEEA, extended end-to-end anastomosis; ESA, end-to-side anastomosis; PAP, patch aortoplasty; SAR, simple aorta releasing; reCoA, recurrent coarctation.

**Figure 2:**
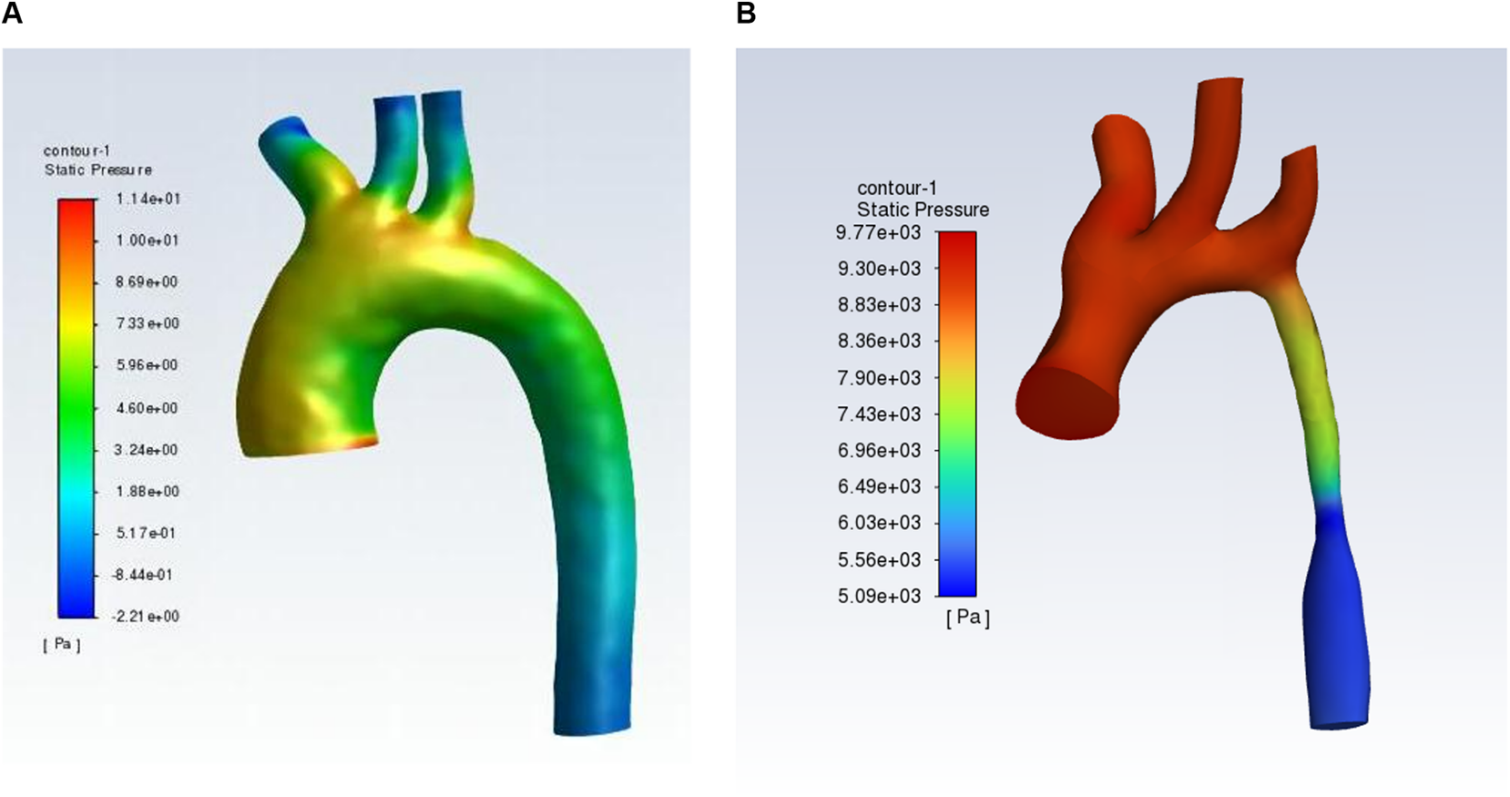
Hydrodynamics shows normal aortic arch **(A)** and coarctation of the aorta **(B)**.

Table 2 shows the relationship between surgical protocol and reCoA. The six surgical regimens were statistically comparable (*P* > 0.05) (Figure 1A). We may hypothesize that selecting the optimal surgical strategy for each case is acceptable since reCoA rates after surgery are not significantly different. Meantime, in certain patients, simple aorta releasing (SAR) may achieve the therapeutic goal (1/2, 50%), highlighting the importance of fluid dynamics even with Artificial Intelligence technology in CoA diagnosis and therapy (Figure 2).

**Table 2:** Relationship between surgical protocol and reCoA.

	EEEA (*n* = 30)	ESA (*n* = 10)	PAP (*n* = 9)	SAR (*n* = 2)	Total (*n* = 51)	*P* value
reCoA, *n* (%)	8 (27)	2 (20)	2 (22)	1 (50)	13 (25)	0.809

The relationship between surgical protocol and reCoA. Values are shown as number (percent %). EEEA, extended end-to-end anastomosis; ESA, end-to-side anastomosis; PAP, patch aortoplasty; SAR, simple aorta releasing; reCoA, recurrent coarctation.

**Figure 3:**
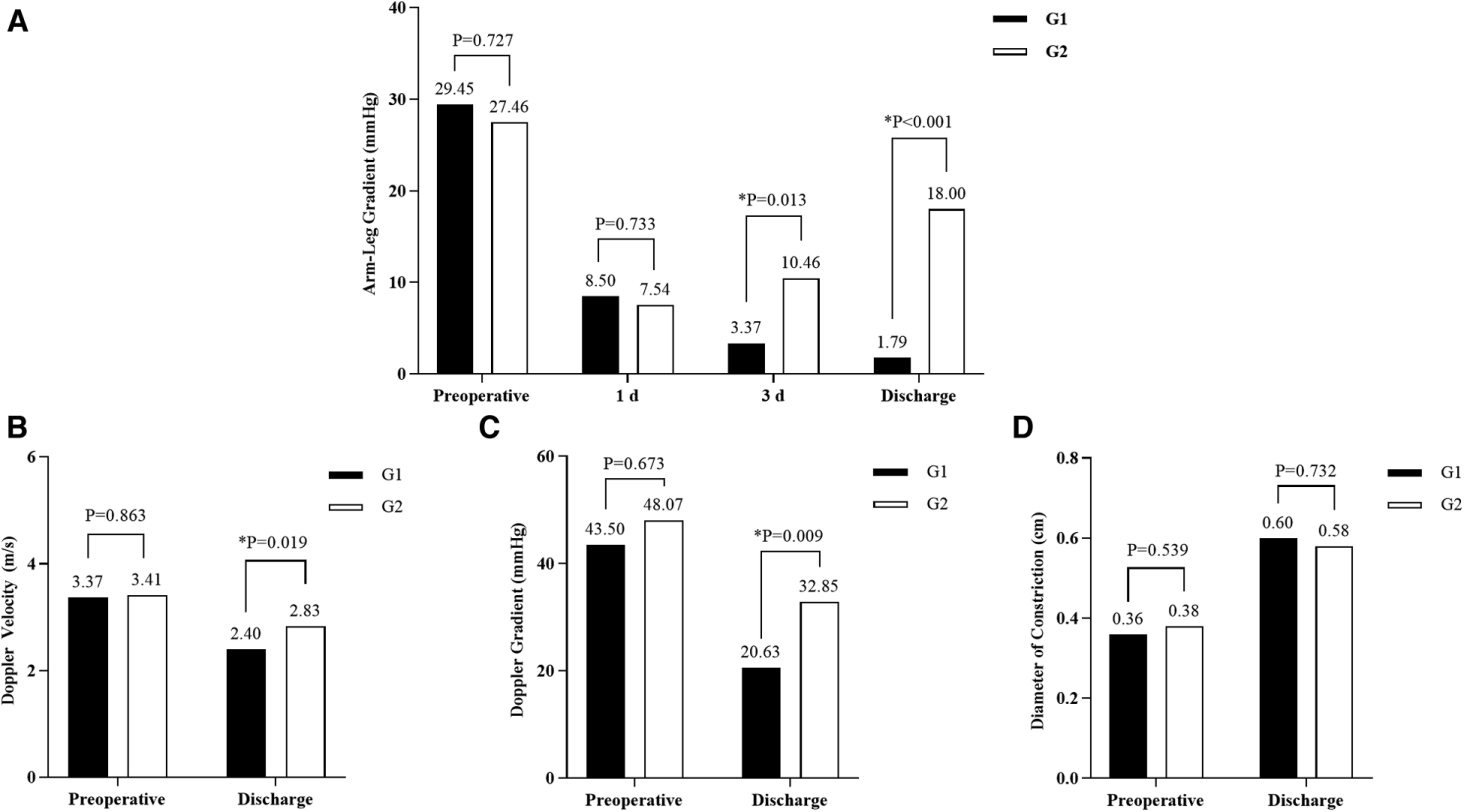
Changes in patient data from postoperative to discharge. Systolic blood pressure gradient (arm-leg) **(A)**. Doppler peak flow velocity across the repair site **(B)**. The Doppler pressure gradient across the repair site **(C)**. Diameter of constriction **(D)**. *Statistical significance.

Figure 3 compares data for different time nodes of G1 and G2. Preoperatively, the mean gradient of G1 was slightly higher than that of G2 (29.45 ± 2.87 vs. 27.46 ± 4.81, *P* = 0.727). Although the mean gradient of G1 was almost identical to that of G2 one day after surgery (8.50 ± 2.49 vs. 7.54 ± 1.30, *P* = 0.733), it was significantly higher in G2 than G1 three days after surgery (10.46 ± 2.06 vs. 3.37 ± 1.45, *P* = 0.013) and at discharge (18, 11.5–21 vs. 1.79 ± 1.12, *P* < 0.001). The gradient of G2 at discharge was about 9 times that of G1 and gradually increased during the period (1 d after surgery, 3 d after surgery, and at discharge), while the gradient of G1 at discharge was lower than that during hospitalization (Figure 3A).

The mean peak Doppler flow velocity at the repair site: G1 was significantly lower than G2 at discharge (2.40 ± 0.09 vs. 2.83 ± 0.14, *P* = 0.019) while almost similar preoperatively (3.37 ± 0.14 vs. 3.41 ± 0.17, *P* = 0.863). Both groups had significantly lower velocity after the operation (G1: *P* < 0.001; G2: *P* = 0.006) (Figure 3B).

G1 had a significantly lower mean pressure gradient at the repair site than G2 at discharge (20.63, 16.24–30.30 vs. 32.85 ± 3.14, *P* = 0.009), which was almost comparable to G2 before surgery (43.50, 32.00–62.25 vs. 48.07 ± 4.52, *P* = 0.673). Gradient made a significant reduction in both groups (G1: *P* < 0.001; G2: *P* = 0.009) (Figure 3C).

The average diameters of repair site in G1 and G2 were almost identical before and after surgery (Preoperative: G1: 0.36 ± 0.02, G2: 0.38 ± 0.03, *P* = 0.539; Postoperative: G1: 0.60 ± 0.03, G2: 0.58 ± 0.03, *P* = 0.732). The diameter increased significantly in both groups at discharge (G1: *P* < 0.001; G2: *P* = 0.001) (Figure 3D).

Use ROC curve to determine which time point between pre-surgery and discharge most affected reCoA and was then included in COX regression. The arm-leg systolic blood pressure gradient, Doppler peak blood flow velocity, and pressure gradient across the repair site (at discharge) were all well affected (Table 3).

**Table 3:** Accuracy in predicting reCoA.

Variable	Arm-leg gradient			Doppler velocity			Doppler gradient		
Time point	AUC	95% CI	*P* value	AUC	95% CI	*P* value	AUC	95% CI	*P* value
Preoperative	0.432	0.25–0.61	0.469	0.554	0.37–0.73	0.567	0.539	0.36–0.72	0.673
1 d	0.432	0.28–0.58	0.469	/			/		
3 d	0.705	0.56–0.85	0.028	/			/		
Discharge*	0.935	0.86–1.00	<0.001	0.740	0.58–0.90	0.010	0.743	0.59–0.90	0.009

Comparison data for different time nodes of G1 and G2. AUC, the area under the curve; CI, confidence interval; reCoA, recurrent coarctation.

*Data at the time point are included for further analysis.

The multivariate COX regression includes variables with *P* < 0.2 in univariate COX regression (Table 4). Among the variable examined, the smaller preoperative z-score of the ascending aorta, the smaller preoperative z-score of the transverse aortic arch, and the higher arm-leg systolic blood pressure gradient at discharge were independent risk factors for reCoA (*P* < 0.05).

**Table 4:** COX regression of reCoA affecting variables.

Variable	*P* value, Exp (b), 95% CI	
	Univariate	Multivariate
Gender: male/female	0.249, 2.14 (0.59–7.76)	
Age at operation	0.137, 1.02 (0.99–1.05)	0.054, 1.04 (1.00–1.07)
Weight at operation	0.101, 1.08 (0.99–1.19)	
Preoperative cardiotonic agents (with or without)	0.173, 0.24 (0.03–1.86)	
Surgery option (radical vs. SAR)	0.042, 0.10 (0.01–0.92)	0.103, 0.01 (0–2.80)
Preoperative ascending aorta, *z*-score	<0.001, 0.59 (0.49–0.71)	0.009, 0.68 (0.51–0.91)
Preoperative transverse aortic arch, *z*-score	<0.001, 0.62 (0.50–0.76)	0.015, 0.66 (0.47–0.92)
Preoperative descending aorta, *z*-score	0.278, 0.84 (0.62–1.15)	
Peak Doppler flow velocity across the repair site at discharge	0.014, 3.23 (1.27–8.24)	
The pressure gradient across the repair site at discharge	0.016, 1.05 (1.01–1.09)	
Arm-leg systolic blood pressure gradient at discharge	<0.001, 1.10 (1.06–1.14)	0.003, 1.09 (1.03–1.15)

The multivariate COX regression includes variables with *P* < 0.2 in univariate COX regression. CI, confidence interval; reCoA, recurrent coarctation.

The Kaplan-Meier curve predicted freedom from reCoA was 70.9% after an average follow-up of 8.93 months (interquartile range, 3.77–19.37 months) (Figure 1B).

The median time from discharge to reCoA was 3.77 months (interquartile range, 1.15–6.32 months). All 13 patients had reCoA within one year, 1 had balloon angioplasty one year after discharge, and 1 is taking antihypertensive agents now, the other 11 patients are currently under close follow-up at the discretion of their parents.

The optimum cutoff for arm-leg systolic pressure gradient at discharge, based on the ROC curve and AUC area, was 12.5 mmHg, with a sensitivity of 76.9% (G2: 10/13) and specificity of 97.4% (G1: 1/38). Thus, patients with a gradient of 12.5 mmHg or higher at discharge had a significantly increased risk of reCoA during follow-up (Figure 1C, log-rank = 49.06, *P* < 0.001).

## Discussion

Coarctation of the aorta (CoA), the sixth most common CHD (13), may occur anywhere in the aorta, mainly distal to the left subclavian artery (14, 15). Morgagni reported CoA in 1760. It could be isolated or related to long-segment stenosis or hypoplastic aortic arch (16). Various CoA treatments now exist, including balloon angioplasty reported by Singer in the 1980s (17). With the decline in surgical mortality, reCoA has received increasing attention with a rate of 5.9–46.6% (18). ReCoA risk factor analysis provides conflicting findings. Young age, low weight, hypoplastic aortic arch, and pressure gradient may increase risk (8–11, 19). In this study, 13 of 51 children developed reCoA within a year, and 1 got balloon angioplasty with satisfactory results. The smaller preoperative *z*-score of the ascending aorta, the smaller preoperative *z*-score of the transverse aortic arch, and the arm-leg systolic blood pressure gradient ≥12.5 mmHg at discharge have an increased risk of reCoA in our patients.

Our research found no significant difference between gender and reCoA (*P* = 0.249), which consists of multiple studies (9, 20, 21). In a study of 167 patients by Burch (10), female showed significant difference (*P* = 0.04, HR = 2.77). 11 females had reCoA (1 Turner's syndrome), which they considered difficult to explain. It has been reported that 7%–12% of Turner syndrome girls in childhood have COA (22). Gene loss on the short arm of the X chromosome may cause isolated CoA (23).

Age and weight at surgery did not increase the risk of reCoA. This finding matched Adamson's study (age, *P* = 0.73) (19). Bacha showed that surgical weight <1.5 kg increased the risk of reCoA (24). It has previously been argued that delaying surgery properly may lower the risk in well-controlled patients. That may be because the aortic arch and constrictions expand steadily as the patient ages, making it easier to detect and remove aberrant areas during operation. With the improved surgical ability and perioperative control, we believe age and weight have lessened their effect on reCoA.

PGE1 has been proven to keep arterial ducts even constrictions open to sustain life. Liberman found ectopic ductal-like tissue in the aorta may induce CoA (25). PGE1 relieves blockage even arterial catheter is closed. Ajay suggested that high-dose PGE1 may treat serious patients with CoA who were unsuccessful in the standard dose (26). However, Burch found that 14 of 105 PGE1-treated patients had reCoA, compared to 1 of 41 non-PGE1-treated patients (*P* = 0.07) (10). We did not use PGE1 preoperatively, thus this factor has not been studied in this study and may be explored in the future. Similar to our findings, reCoA has not been connected to cardiac abnormalities (8, 9, 19, 27).

We believed preoperative cardiotonic agents do not increase the risk of reCoA. But Truong found that did (*P* = 0.04, HR = 5.57), while multivariate analyses did not (12). We hypothesized that probably because most young PGE1 recipients were treated with cardiotonic agents preoperatively.

Many studies have examined how surgery option affects reCoA. Several procedures have been developed to reduce risk. However, the optimal one is still debated (28, 29). Crafoord proposed EEA in 1944 (3), despite a decreased mortality (6), the reCoA rate was high (4–6), probably because of incomplete resection of the catheter tissue, which partially grow into the normal-appearing aorta wall, lack of growth at the circular anastomosis and the hypoplastic aortic arch. Thus, patch aortoplasty has gradually replaced EEA (30). Patches materials include artificial materials, allogeneic blood vessels, autologous pulmonary artery, or pericardium. The anastomotic stoma is tension-free, collateral vessels do not need to be ligated or disconnected, and the hypoplastic region of the arch can be extended at the same time. But Adamson linked patch material to reCoA (*P* = 0.014, OR = 9.26) (19), and long-term patch's contralateral aortic posterior wall aneurysm is another potential risk (31). EEEA can better manage remaining catheter tissue, protect the left subclavian artery, avoid artificial materials, retain natural vascular architecture, lower aneurysm risk, and repair transverse arch and isthmus dysplasia (32). Meantime, EEEA reduces mortality and reCoA rate (33, 34) and promotes long-term aortic compliance (35), so it may be the best potential surgery. Patients with other CHD may accept one-period surgery through median sternal, with satisfactory outcomes (36, 37). Results from the analysis of ESA were similar (33, 37, 38). Balloon angioplasty can cure reCoA well (10, 17, 39), with a 93% success rate (40). One reCoA patient in this study got balloon angioplasty without any additional intervention.

Each strategy has pros and cons. EEEA or ESA may cause patients to suffer from large-scale surgery, which is psychologically and financially taxing. Nearly 60% of patients in this research had successful EEEA. Meantime, if we took EEEA, ESA, and PAP as radical surgery whereas SAR was considered a relative surgery. Neither was proven to be reCoA risk factor (*P* = 0.075). Although fewer patients had SAR, we assumed that careful selection of operation according to ease patients may even prevent major surgery. Therefore, in the future, we may take more in-depth research on the morphology and hydrodynamics of the aortic arch during the perioperative period, and even pre-do the operation with Virtual Reality (VR), to allow surgeons to better determine the therapy for patients.

Multiple studies have explored aortic arch morphology and whether systolic blood pressure gradient affects reCoA. We found that the preoperative *z*-score of the ascending aorta impacted reCoA (*P* = 0.009), which matched Kumar (34). In this 10-variable research, reCoA was only associated with a small preoperative ascending aorta (*P* < 0.01). McElhinney agreed (*P* = 0.02, HR = 2.1), but disagreed when body weight was the indicator (*P* = 0.48, HR = 1.34) (9). This may be because children with lower body weight in cohorts of patients had a lesser ascending aorta. Unfortunately, maybe because of the limited sample size in the cohort, we could not identify an optimal cutoff for the ascending aorta z-score linked with reCoA. Future research in larger cohorts is conceivable.

A smaller preoperative z-score of the transverse aortic arch increased the risk of reCoA (*P* = 0.015). Burch found that for every 1 mm increase in aortic arch transverse diameter, reCoA risk was reduced (*P* = 0.04, RR = 0.57) (10). However, Kumar (34) cannot relate transverse arch dysplasia to reCoA. Truong found that preoperative aortic arch measurements and transverse aortic arch abnormalities were not reCoA risk factors in thoracotomy patients (12). We believe our findings are reliable, cardiac surgeons should choose the approach carefully for patients with smaller preoperative z-score of the transverse aortic arch to improve their outcomes.

Arm-leg systolic blood pressure gradient ≥12.5 mmHg at discharge affected reCoA (*P* < 0.001, log-rank = 49.06). Kumar examined blood pressure gradients at 24 h, 48 h, and 72 h following surgery and at discharge, finding that the gradient at discharge was significant compared with other points (34), reCoA was more likely in patients with a gradient >13 mmHg (*P* < 0.001, log-rank = 19.49). Although we considered that in the early postoperative stage, blood pressure was unstable due to operation, anesthesia, and other factors, we think the conclusion is reliable. In the future, more precise pressure measurements can help further explore its relationship with reCoA.

About 60% (8/13) developed reCoA within 6 postoperative months, and all within one year. Therefore, we suggested that closer follow-up is necessary for the first year postoperatively.

### Limitations

This work is unusual because we evaluated the impacts of several parameters on reCoA and found the blood pressure cutoff and its specificity and sensitivity to better predict reCoA, which has great practical application. However, our study had several limitations. This is a retrospective study that has the inherent limitations of any retrospective study. Five congenital cardiac surgeons operated the surgical procedures and at least three different echocardiography specialists were included to take the measurements of aortic arch morphology preoperatively and postoperatively. Meanwhile, several issues need to be explored, including the best preoperative z-score cutoff of the ascending aorta and transverse aortic arch, which may be examined in a larger sample cohort. For reCoA, because the follow-up did not exactly follow the plan, the accurate reCoA time may be earlier, but all reCoA can be found within 1 year after the surgical procedure, so we think it does not affect the study results.

## Conclusion

In conclusion, the smaller preoperative *z*-score of the ascending aorta, the smaller preoperative *z*-score of the transverse aortic arch, or the discharge arm-leg systolic blood pressure gradient ≥12.5 mmHg make an increased risk of reCoA. We suggested more active follow-up for such patients, especially within 1 postoperative year, to detect reCoA timely.

## Data Availability

The raw data supporting the conclusions of this article will be made available by the authors, without undue reservation.
